# A Potential Link Between Dyslipidemia and Inflammation in Patients with Schizophrenia: Insights from Sterols and HDL Profile Analysis

**DOI:** 10.3390/ijms27167219

**Published:** 2026-08-13

**Authors:** Sveva Bagnasco, Giuseppe De Simone, Gustavo Cernera, Alessandro Usiello, Felice Iasevoli, Giuseppe Castaldo, Monica Gelzo, Andrea de Bartolomeis

**Affiliations:** 1 CEINGE-Biotecnologie Avanzate Franco Salvatore, 80145 Naples, Italy; bagnasco@ceinge.unina.it (S.B.); gustavo.cernera@unina.it (G.C.); alessandro.usiello@unicampania.it (A.U.); giuseppe.castaldo@unina.it (G.C.); 2 Dipartimento di Medicina di Precisione in Area Medica, Chirurgica e Critica, Università di Palermo, 90127 Palermo, Italy; 3 Sezione di Psichiatria, Laboratorio di Psichiatria Traslazionale e Molecolare e Unità di Psicosi Resistente al Trattamento, Dipartimento di Neuroscienze, Scienze della Riproduzione e Odontostomatologia, Università di Napoli Federico II, 80131 Naples, Italy; giuseppe27.desimone@gmail.com (G.D.S.); felice.iasevoli@unina.it (F.I.); adebarto@unina.it (A.d.B.); 4 Dipartimento di Medicina Molecolare e Biotecnologie Mediche, Università di Napoli Federico II, Via Pansini 5, 80131 Naples, Italy; 5 Dipartimento di Scienze del Farmaco per l’Ambiente e la Salute, Università degli Studi della Campania Luigi Vanvitelli, 81100 Caserta, Italy

**Keywords:** cholesterol, HDL, schizophrenia, inflammation

## Abstract

Dyslipidemia and inflammation are two hallmarks of schizophrenia (SCZ), but the link between these two conditions remains elusive. We studied 40 SCZ patients and 40 age- and sex-matched healthy controls (CTRL) analyzing serum total, low-density lipoprotein (LDL), and high-density lipoprotein (HDL) cholesterol. In addition, we evaluated, for the first time in SCZ patients, HDL subfractions and surrogate markers of cholesterol intestinal absorption and de novo synthesis together with cholesterol precursors, aiming to address a possible dysregulation of cholesterol metabolism in SCZ. Moreover, we measured serum cytokines to evaluate systemic inflammation. SCZ patients showed an impaired cholesterol absorption, as indicated by serum phytosterols and cholestanol reduction, and a subsequent increase in de novo synthesis, confirmed by the increase in serum lathosterol. However, the last step of cholesterol biosynthesis seems to be impaired, probably for the inhibition of the 7-dehydrocholesterol reductase (DHCR7), as suggested by the significant increase in serum 7-dehydrocholesterol (and its isomer 8-dehydrocholesterol). Consequently, serum HDL cholesterol is significantly reduced with an anti-inflammatory remodeling of its subfractions that likely represents a response to systemic inflammation suggested by the increased serum interleukin-6 and angiopoietin-2 levels. Overall, the impaired cholesterol metabolism in SCZ patients is associated with a pro-inflammatory state, which is responded to by the anti-inflammatory remodeling of HDL subfractions. Improving intestinal cholesterol absorption and reducing endogenous cholesterol biosynthesis could represent putative therapeutic approaches.

## 1. Introduction

Schizophrenia (SCZ) is a severe, clinically heterogeneous psychiatric disorder affecting approximately 1% of the global population, usually diagnosed in adolescents or young adults and is conceptualized as a disorder of synapse and cortico-subcortical connectivity [1]. Genetic predisposition and environmental factors increase the risk of SCZ, but its pathogenesis is still under investigation [2]. Brain neurotransmitter imbalance (involving monoaminergic, glutamate, and GABA systems) is a hallmark of SCZ and antipsychotics, the mainstay of pharmacological treatment, share the common feature of occupying dopamine D2 receptors. Alterations involving lipid metabolism and inflammation have been reported in SCZ patients [3] and are presumably related to the pathophysiology of SCZ as well as to the pharmacological treatment. Specifically, abnormal serum levels have been detected for cholesterol that is a crucial molecule for the brain structure and function, since brain contains up to 20% of the entire human body cholesterol, and is mostly independent of systemic cholesterol metabolism, as the blood-brain barrier (BBB) is impermeable to cholesterol [4]. Altered levels of low-density lipoprotein (LDL) cholesterol, high-density lipoprotein (HDL) cholesterol and triglycerides in serum have been found in patients with schizophrenia [3], correlating with the clinical expression of the disease with conflicting results. For example, an increase in HDL cholesterol has been correlated with the expression of negative symptoms, but without a clear significance regarding the degree and extent of symptomatology [5,6]. Furthermore, the anti-inflammatory role of HDL cholesterol is known [7], but in other studies a triggering role for neuroinflammation has been found [8]. This discrepancy may be due to a different serum distribution of HDL subclasses, with pro- or anti-inflammatory properties [9], which have not been studied so far in patients with SCZ.

Furthermore, alterations in lipid metabolism may be caused by the drugs used to treat patients with SCZ. For example, olanzapine contributes to hypertriglyceridemia and hepatic steatosis [10], while most antipsychotic drugs cause an increase in serum cholesterol, LDL, and triglycerides and a decrease in HDL [11]. Finally, other antipsychotics inhibit the last enzyme in the cholesterol synthesis pathway, causing the accumulation of biosynthetic intermediates such as 7- and 8-dehydrocholesterol (DHC), which can produce oxysterols and other toxic neurosterols [12,13].

To cast light on the relationship between lipid metabolism and inflammation, we studied 40 patients with SCZ and 40 healthy control subjects, matched for age and sex, analyzing serum levels of total cholesterol, LDL and HDL, HDL subclasses, cytokines, surrogate markers of intestinal absorption and de novo biosynthesis, together with cholesterol precursors.

## 2. Results

We studied 40 patients with SCZ in comparison with 40 age- and sex-matched healthy controls (CTRL). Demographic and clinical characteristics of the study population are described in Table 1. All patients were under pharmacological treatment with antipsychotics of both the first and second generation; 21 patients responded to the therapies (noTRS) and the remaining 19 were defined as TRS (treatment-resistant schizophrenia) patients (Table S1).

**Figure 1:**
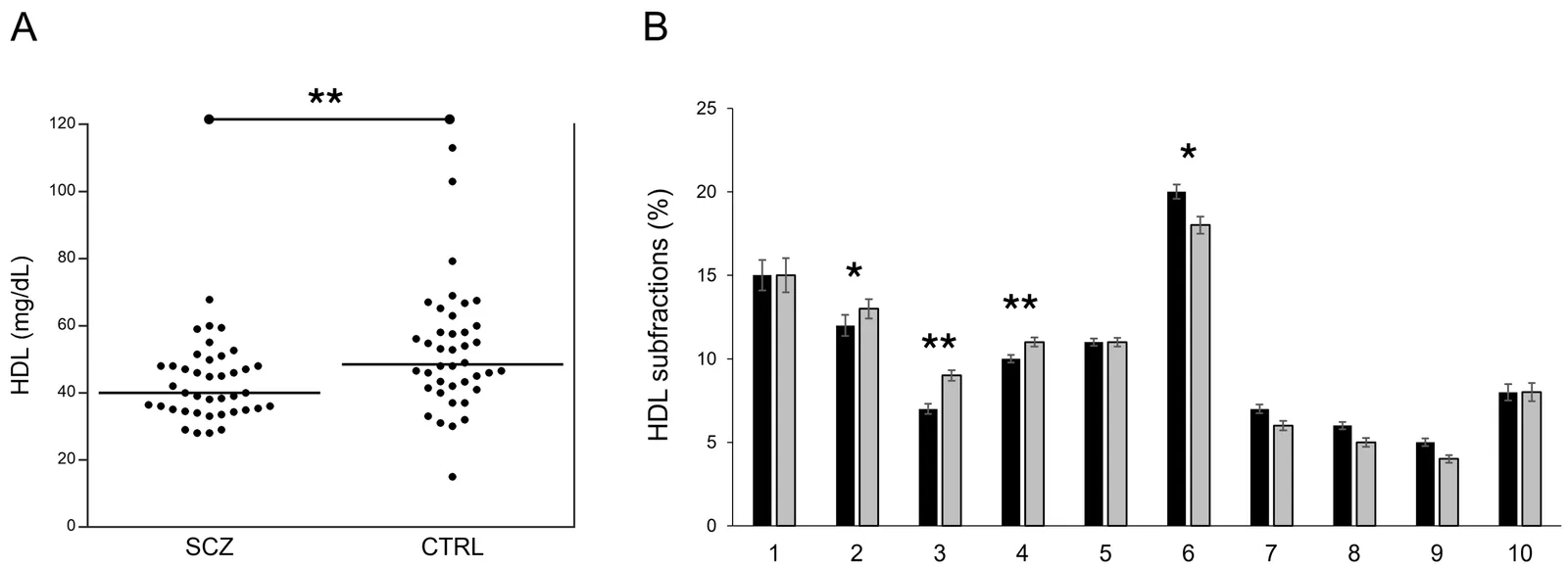
Serum HDL cholesterol (**A**) and HDL subfractions % (**B**) in 40 patients with schizophrenia (SCZ) and in 40 sex- and age-matched healthy controls (CTRL). In (**B**), black and gray bars represent the median levels of each HDL subfraction % in SCZ patients and CTRL, respectively; error bars correspond to standard error. *: *p* < 0.05, **: *p* < 0.005.

We analyzed serum lipids in SCZ patients and in CTRL. Total and LDL cholesterol were not significantly different between the two groups, while HDL cholesterol levels were significantly lower in SCZ patients than in CTRL (Table 2 and Figure 1A). Serum triglycerides levels were significantly higher in SCZ patients, whereas albumin levels were not significantly different between the two groups.

**Figure 2:**
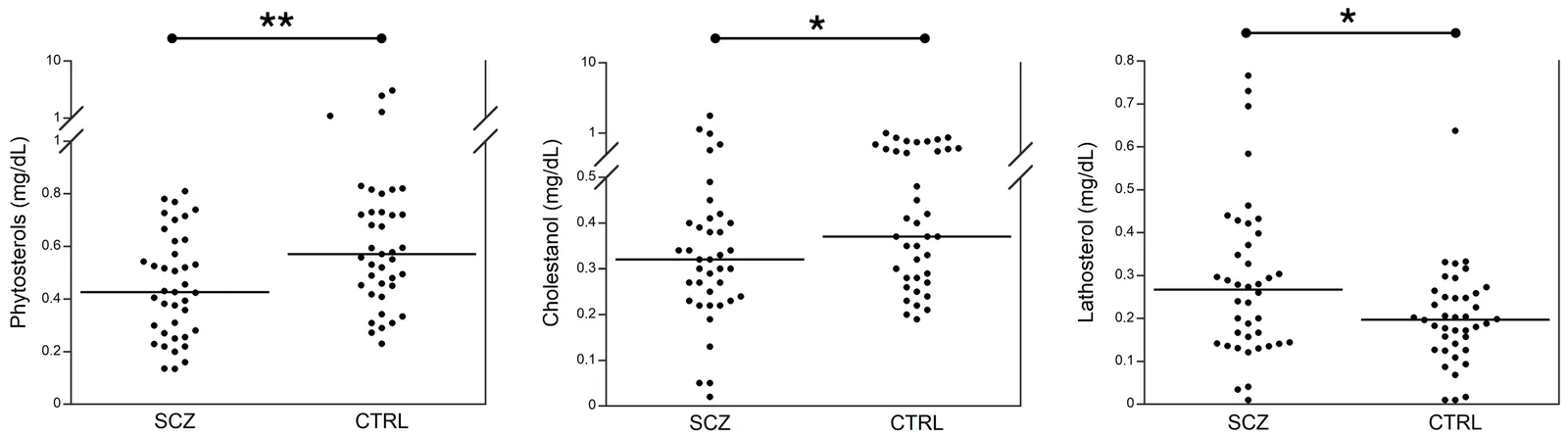
Serum phytosterols, cholestanol, and lathosterol in 40 patients with schizophrenia (SCZ) and in 40 sex- and age-matched healthy controls (CTRL). *: *p* < 0.05, **: *p* < 0.005.

Therefore, we analyzed serum surrogate markers of cholesterol absorption (i.e., phytosterols and cholestanol) and de novo synthesis (i.e., lathosterol). As shown in Table 2 and Figure 2, serum phytosterols as well as cholestanol levels were significantly lower in SCZ patients as compared to CTRL, while serum lathosterol was significantly higher.

**Figure 3:**
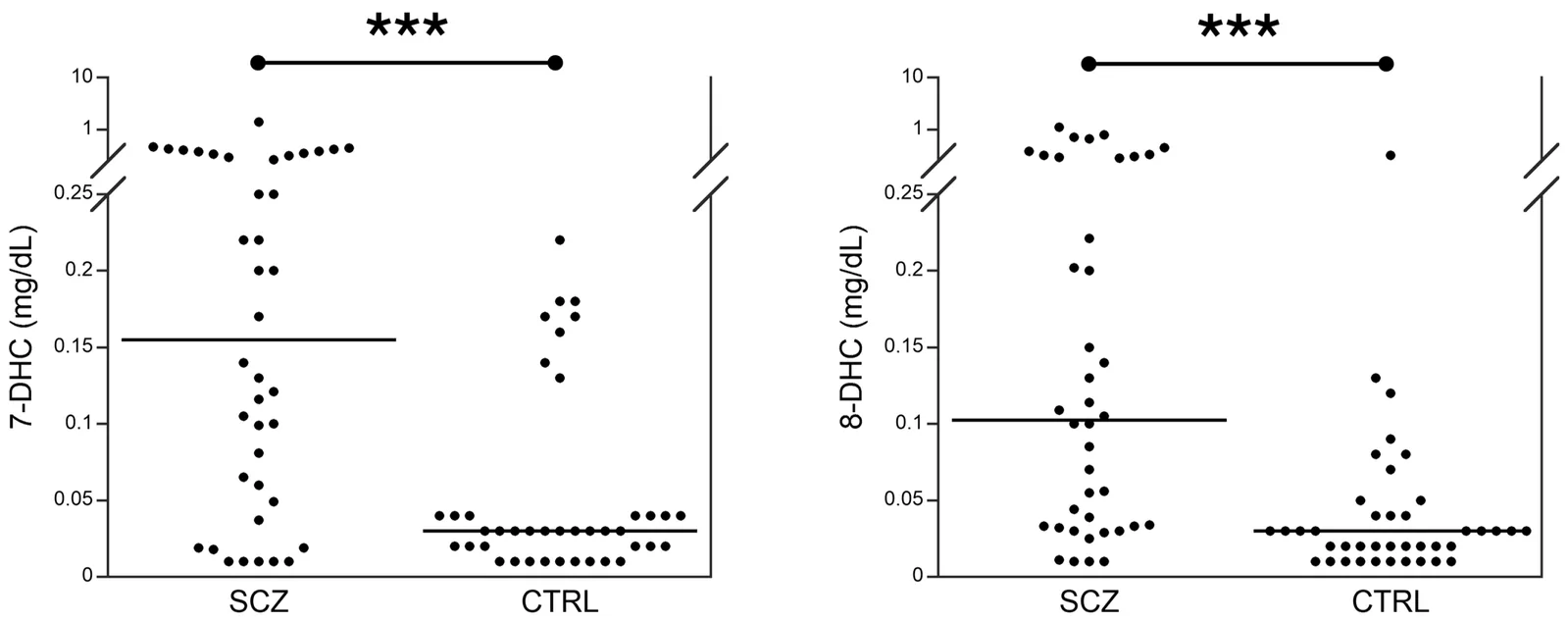
Serum 7- and 8-dehydrocholesterol (DHC) in 40 patients with schizophrenia (SCZ) and in 40 sex- and age-matched healthy controls (CTRL). ***: *p* < 0.001.

In addition, we analyzed serum levels of all cholesterol precursors, and 7- and 8-DHC levels were significantly higher in patients with SCZ than in CTRL, while serum desmosterol was not significantly different (Table 2 and Figure 3). Thirteen out of 40 SCZ patients had serum levels of 7-DHC > 0.25 mg/dL which represent the upper value in CTRL; among these 13 patients, five were in treatment with haloperidol, five with aripiprazole, two with clozapine and one with other drugs (Table S1). Among the other 27 patients, only two were in treatment with haloperidol and four with aripiprazole.

Furthermore, we analyzed serum HDL subfractions in the 40 patients with SCZ and the corresponding CTRL. As shown in Figure 1B, the percentages relative to total HDL for the HDL 2, 3, and 4 subfractions were significantly lower in SCZ patients than in CTRL, while the percentage of the HDL 6 subfraction was significantly higher in SCZ.

**Figure 4:**
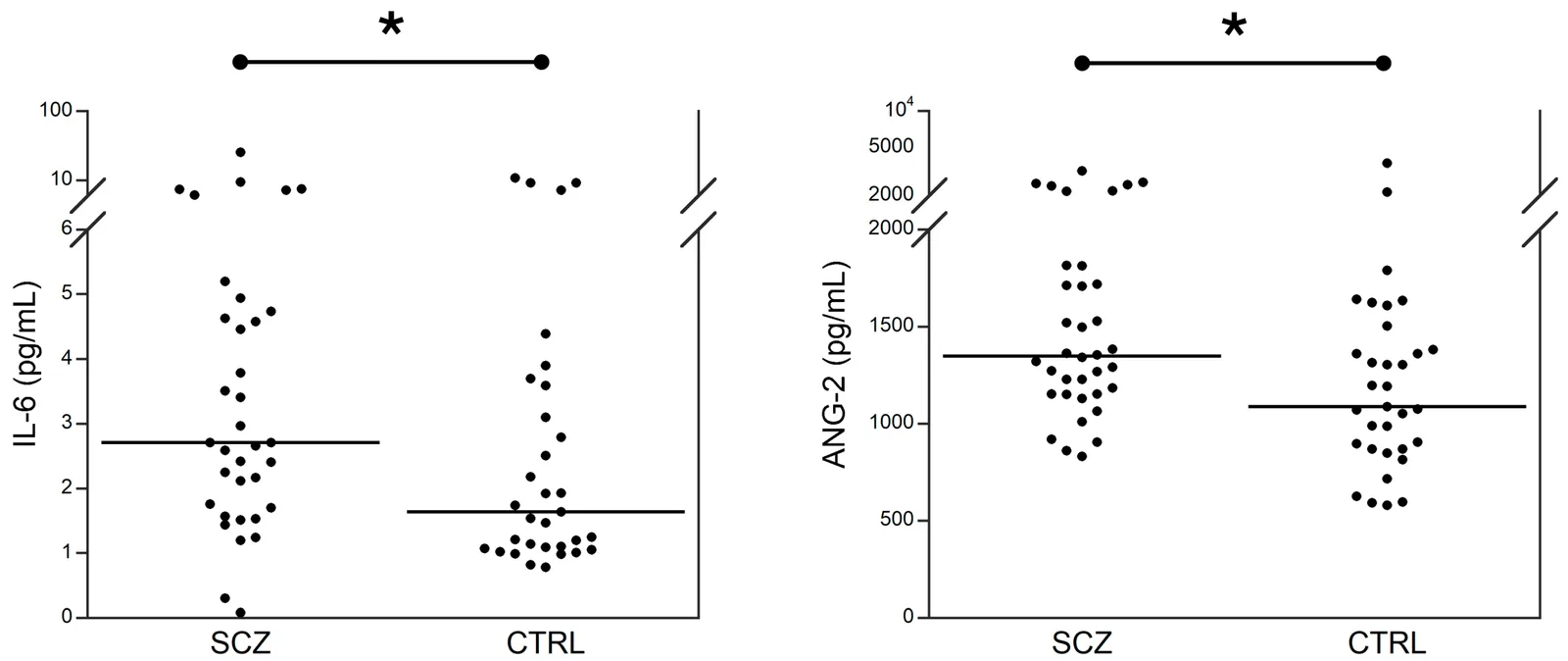
Serum interleukin (IL)-6 and angiopoietin (ANG)-2 in 40 patients with schizophrenia (SCZ) and in 40 sex- and age-matched healthy controls (CTRL). *: *p* < 0.05.

We then measured serum cytokines, and we found significantly higher levels of angiopoietin (ANG)-2 and interleukin (IL)-6 in SCZ patients than in CTRL (Table 3 and Figure 4).

Moreover, we measured blood cell counts and fibrinogen levels in SCZ patients (Table 3). Although median values of these markers are within our normal ranges, we found that 3/40 patients had neutrophil counts higher than 7500 cells/µL and 8/40 patients showed NLR values higher than 3.0.

We also compared lipids and inflammation markers between TRS and noTRS subgroups (Table S2). No significant differences were observed between the two groups, except for serum IL-10 that was significantly higher in TRS than noTRS (2.30 pg/mL vs. 1.94 pg/mL, *p* = 0.019).

Subsequently, we evaluated the correlations between discriminant lipids (i.e., markers that were significantly different between SCZ and CTRL groups) and serum cytokines in SCZ patients (Table 4). We found a significant negative association between HDL cholesterol and VEGF-A, while 7-DHC and 8-DHC were positively associated with VEGF-A and ANG-2, respectively.

Finally, we performed a linear regression analysis between antipsychotic doses (expressed as chlorpromazine equivalents, CPZeq) and the discriminant variables in SCZ patients (i.e., markers that were significantly different between SCZ and CTRL groups), including also IL-10, to determine whether these significant differences were attributable to antipsychotic therapy or to the disease itself. As shown in Table 5, the serum levels of 8-DHC and IL-10 were positively related to CPZeq.

We also evaluated the correlations between all lipid markers and the three PANSS values, i.e., positive, negative and general, but no significant associations have been observed.

## 3. Discussion

Patients with SCZ showed an impaired absorption of cholesterol, as indicated by the reduction in serum phytosterols and cholestanol that are surrogate markers of cholesterol absorption [14], and a subsequent increase in de novo synthesis of cholesterol, confirmed by the increase in serum lathosterol, a surrogate marker of cholesterol biosynthesis [14]. However, the last step of cholesterol biosynthesis is impaired, as indicated by the increase in the last precursors, i.e., 7-DHC (and its isomer 8-DHC) [15], likely for the inhibition of the DHCR7. Consequently, serum HDL cholesterol is significantly reduced, but there is a remodeling of serum HDL subfractions with a significant reduction in pro-inflammatory large HDL [16] together with an increasing trend of anti-inflammatory small HDL [17]. Likely, such remodeling represents a response to systemic inflammation suggested by the increased levels of serum IL-6 and ANG-2 and is probably due to a potential enhanced synthesis of pro-inflammatory oxysterols from the 7- and 8-DHC substrates [18].

The study of serum lipids in patients with SCZ is a relevant topic of research for the impact on the clinical expression of the disease, but conflicting results have been reported so far. For example, a study on 55 SCZ patients reported a significant increase in serum triglycerides and the significant reduction in HDL [19] in agreement with us, while another study on 78 SCZ patients reported a significant increase in triglycerides, found also in our patients, and in LDL and cholesterol [20] that we did not find. Moreover, an increase in HDL was reported in SCZ patients with less negative symptoms [5] but also in women with negative symptoms [6]. In addition, while the anti-inflammatory role of HDL in SCZ is known [7], a trigger role for neuroinflammation has been found in another study that demonstrated the HDL shuttling of endotoxins to the brain [8]. A reason for such conflicting results may be the different profile of HDL subfractions in serum. Small HDL (i.e., HDL 8–10) are the native form of the molecule, which are converted into intermediate (i.e., HDL 4–7) and large (i.e., HDL 1–3) HDL [21]. Small HDL, richer in cholesteryl esters, display an anti-inflammatory effect while large ones, enriched in unesterified cholesterol and triglycerides, display a pro-inflammatory role. The impaired de novo synthesis of cholesterol, which we observed in our SCZ patients, and the subsequent altered reverse cholesterol transport causing a reduction in HDL in serum are the picture of a pro-inflammatory response that we already observed in a multi systemic inflammatory syndrome of children [9]. On the other hand, systemic inflammation, which involves brain structures implicated in the development of SCZ and other neuropsychiatric disorders [22], and inflammatory biomarker findings, including HDL-based inflammatory indices [7], revealed inflammation as a hallmark of SCZ patients, which can also modulate the responsiveness to treatments [23]. Interestingly, we observed a reduction in pro-inflammatory HDL subfractions 2–4, which could be a positive response to the reduced reverse transport of HDL.

Some of the alterations that we observed in serum could be mirrored at the brain level in an animal model of the disease. In fact, a mice model of SCZ showed an increase in 7-DHC due to impaired cholesterol biosynthesis in the striatum and prefrontal brain cortex also with gender differences [13]. 7-DHC is an unstable and toxic [24] metabolite, which is rapidly converted by autoxidation into toxic oxysterols [25], vitamin D, and atypical neurosteroids [12] which cause myelination impairment, extrapyramidal effects and autistic manifestations in up to 70% of cases [12]. On the other hand, oxysterols, unlike cholesterol, cross the BBB [26], and brain oxysterols may play a pivotal role in SCZ development in risk subjects [26]. Interestingly, we observed that the levels of serum desmosterol in patients with SCZ were not significantly different as compared to CTRL, differing from a previous study on mice that reported reduced serum levels of desmosterol [13]. Our data indicate that despite DHCR7 inhibition, the synthesis of cholesterol at the brain level is maintained.

We also analyzed a panel of serum cytokines, and we found significantly higher levels of IL-6 and ANG-2 in SCZ patients that suggest a systemic inflammatory status. In particular, ANG-2 has been implicated in the regulation of inflammatory responses [27] and has been reported to be elevated in a subgroup of SCZ patients characterized by altered circulating growth factor profiles [28]. Inflammatory cytokines can induce the expression of ANG-2, which can antagonize angiopoietin-1, thereby disrupting vascular integrity and increasing endothelial permeability. These mechanisms suggest that elevated ANG-2 may contribute to vascular dysfunction and inflammation, processes that have been increasingly implicated in the pathophysiology of SCZ [29].

Moreover, we found positive correlations of serum 7-DHC and 8-DHC levels versus VEGF-A and ANG-2. Since Ang-2 induces vascular destabilization, allowing VEGF-A to drive endothelial plasticity [30], 7-DHC and 8-DHC may be involved in the structural remodeling of vessels as well as in pro-inflammatory processes. At the same time, HDL cholesterol is inversely correlated with serum VEGF-A levels, thus suggesting an antagonistic role towards all these effects.

This study has limitations that should be acknowledged. First, the small number of subjects included in the analysis may have hampered the precise dissection of all putative significant differences in lipid metabolites between SCZ patients and CTRL. Second, alterations of lipid metabolism in patients with SCZ may also be due to pharmacological treatment. For example, it is known that olanzapine contributes to hypertriglyceridemia and liver steatosis [10] and the same is true for most of the antipsychotic drugs of the second generation used in the therapy of patients with SCZ [11]. Other drugs, both antagonist and partial agonist of dopamine such as haloperidol and aripiprazole [12], interfere with de novo biosynthesis of cholesterol, inhibiting DHCR7. Among the 13 SCZ patients with 7-DHC levels > 0.25 mg/dL, 10 were in treatment with such drugs. Similarly, in several patients treated with olanzapine and clozapine, 7-DHC levels results were higher than CTRL, suggesting that some impairment of cholesterol biosynthesis may also occur in SCZ patients not treated with drugs impairing cholesterol biosynthesis [12,13]. However, the changes reported after chronic drug treatment did not match the ones that we reported and possibly cannot be ascribed to drug treatment only. Furthermore, we performed linear regression analysis to evaluate the potential impact of antipsychotic dosage (expressed as CPZ equivalents) on the levels of the measured analytes. Only 8-DHC levels were positively related to CPZeq, although with a borderline *p*-value. Therefore, we cannot state with certainty that the alterations observed for 8-DHC levels in SCZ patients are attributable to antipsychotic treatment—particularly in the case of 7-DHC, which showed no association with drug dosages.

To conclude, our study suggests an altered intestinal absorption and impaired endogenous biosynthesis of cholesterol in SCZ patients with the coexistence of pro-inflammatory processes and anti-inflammatory processes (represented by the remodeling of HDL subfractions). Further studies, especially longitudinal, are needed to capture the full relevance of serum lipid alterations on the clinical expression of SCZ, but our data, although preliminary, suggest that the improvement of cholesterol absorption and the modulation of de novo biosynthesis might represent a contributory approach in patients with SCZ.

## 4. Materials and Methods

### 4.1. Patients

We enrolled 40 patients with SCZ diagnosis and 40 age- and sex-matched CTRL who have never undergone psychiatric or metabolic treatments. The study was approved by the Ethics Committee of Federico II University Hospital (protocol number: 195/19) and was performed according to the current version of the Helsinki Declaration. Written informed consent was obtained from all patients and healthy volunteers enrolled. The diagnosis of SCZ has been performed according to the criteria of The Diagnostic and Statistical Manual of Mental Disorders-5th edition [31]. Inclusion criteria for patients were: age 18–60 years; no evidence of worsening psychotic symptoms in the previous 6 months; absence of other major systemic, psychiatric (e.g., addictive disorders, frequent substance use in the 6 months prior to enrollment, etc.), or neurological disorders. Psychotic symptoms were assessed through three dimensions of the Positive and Negative Syndrome Scale (PANSS) [32], the Positive Syndrome Scale, the Negative Syndrome Scale and the General Psychopathology Subscale.

TRS condition was defined as a failure of at least two different antipsychotic regimens, each administered for >6 weeks and at an optimal dose, according to the modified Treatment Response and Resistance in Psychosis Working Group Consensus criteria [33].

### 4.2. Biochemical Analyses

Blood samples were collected in plastic tubes without anticoagulant/additive and centrifuged at 3500 rpm for 15 min at 4 °C. Serum samples were collected and stored at −80 °C. Serum total, HDL and LDL cholesterol, and triglyceride levels were measured by the Cobas prointegrated system (Cobas ISE, Cobas c503, Cobas e801, Roche Diagnostics, Zug, Switzerland) using specific commercial kits (Roche Diagnostics). Blood cell count was performed on EDTA-anticoagulated whole blood using the Advia 2120i hematology system (Siemens, Munich, Germany). Fibrinogen was measured on sodium citrate-anticoagulated whole blood by ACL TOP 750 CTS (Werfen, Barcelona, Spain).

### 4.3. Serum Cytokines

Serum IL-5, IL-6, IL-8, IL-10, IL-13, IL-33, ANG-2, tumor necrosis factor (TNF)-α, interferon (IFN)-γ, and vascular endothelial growth factor A (VEGF-A) were analyzed by automated microfluidic immunoassay cartridges on ProteinSimple Ella (Bio-Techne, Minneapolis, MN, USA), according to the manufacturer’ instructions [9].

### 4.4. Sterol Profile Analysis

The sterol analysis has been performed as previously described [34,35]. Briefly, 50 μL of serum were mixed with 40 μg of 5α-cholestane (internal standard) and hydrolyzed by incubation in 1 N potassium hydroxide ethanolic solution at 80 °C for 1 h. The sterols were then extracted by hexane and derivatized by N,O-bis(trimethylsilyl)trifluoroacetamide and pyridine, dried under nitrogen, and dissolved in 100 μL of dichloromethane. For qualitative and quantitative sterol analysis, 1 μL of solution was injected into a gas chromatograph coupled with a mass spectrometer (GC-MS; 8860 GC, 5977C GC/MSD, Agilent Technologies, Santa Clara, CA, USA).

### 4.5. HDL Subfraction Analysis

The analysis of HDL subfractions was performed by the Quantimetrix kit (Quantimetrix Corp., Redondo Beach, CA, USA). In brief, serum (25 μL) was added to the precast high-resolution polyacrylamide gel tubes along with Lipoprint HDL Loading Gel solution (300 μL). The tubes were photopolymerized and subjected to electrophoresis. Then, the tubes were scanned and analyzed using the Lipoprint LDL/HDL subfractions system. The ten HDL subfractions were differentiated by polyacrylamide gel electrophoresis using the Quantimetrix kit (Quantimetrix Corp.). The HDLs were separated on the base of their size into 10 subfractions, which were subsequently grouped into three main classes: large (from HDL-1 to HDL-3), intermediate (from HDL-4 to HDL-7) and small (from HDL-8 to HDL-10) [9].

### 4.6. Statistical Analysis

Categorical data are reported as frequency (percentage) and compared by chi square test. Continuous data are reported as median (interquartile range, IQR) and compared by Mann–Whitney U test. The Shapiro–Wilk test was applied to evaluate the normality of distributions. Spearman correlation analysis was applied to evaluate associations between serum lipids and cytokines. To test the association of discriminant variables vs. CPZeq, a linear regression analysis with discriminant variables as dependent variables has been performed. Statistical analyses were performed by SPSS (version 30, IBM SPSS Statistics, Armonk, NY, USA). Graphics were created by KaleidaGraph software (version 4.5.4, Synergy, Reading, PA, USA).

## Figures and Tables

**Table 1:** Demographic and clinical characteristics of the study population.

Parameters	SCZ (n = 40)	CTRL (n = 40)
Female, n (%)	12 (30)	12 (30)
Age (years)	38 (30–52)	38 (29–51)
Diagnosis age (years)	21 (18–27)	-
TRS, n (%)	19 (47.5)	-
PANSS-POS	20 (14–23)	-
PANSS-NEG	24 (19–27)	-
PANSS-GEN	44 (39–52)	-

SCZ: schizophrenia; CTRL: healthy controls; TRS: treatment-resistant schizophrenia; PANSS: positive and negative syndrome scale; POS: positive; NEG: negative; GEN: general.

**Table 2:** Comparison of serum lipids between patients with schizophrenia (SCZ, n = 40) and healthy controls (CTRL, n = 40).

Serum Lipids (mg/dL)	SCZ	CTRL	*p*-Value
Total cholesterol	163 (139–188)	164 (141–180)	0.675
LDL cholesterol	84 (64–101)	92 (73–106)	0.235
HDL cholesterol	40 (35–48)	49 (42–60)	**0.004**
Triglycerides	120 (86–157)	91 (67–115)	**0.005**
Lathosterol	0.27 (0.14–0.40)	0.20 (0.14–0.26)	**0.035**
Phytosterols	0.43 (0.27–0.62)	0.57 (0.45–0.73)	**0.003**
Cholestanol	0.32 (0.23–0.40)	0.37 (0.28–0.59)	**0.042**
Albumin	4.58 (4.22–4.95)	4.46 (4.08–4.73)	0.491
7-DHC	0.17 (0.06–0.34)	0.03 (0.02–0.04)	**<0.001**
8-DHC	0.11 (0.03–0.29)	0.03 (0.02–0.04)	**<0.001**
Desmosterol	0.12 (0.05–0.24)	0.16 (0.05–0.24)	0.787

LDL: low-density lipoprotein; HDL: high-density lipoprotein; DHC: dehydrocholesterol. Significant *p*-values are reported in bold.

**Table 3:** Comparison of inflammation markers between patients with schizophrenia (SCZ, n = 40) and healthy controls (CTRL, n = 40).

Markers	SCZ	CTRL	*p*-Value
IL-13 (pg/mL)	3.23 (1.60–8.40)	2.85 (1.50–12.75)	0.714
ANG-2 (pg/mL)	1349 (1053–1789)	1089 (870–1443)	**0.009**
TNF-α (pg/mL)	10.2 (9.0–12.0)	9.42 (7.49–10.70)	0.058
IL-10 (pg/mL)	2.06 (1.68–2.85)	1.88 (1.46–2.51)	0.339
IL-6 (pg/mL)	2.71 (1.70–4.74)	1.64 (1.08–3.65)	**0.026**
IL-5 (pg/mL)	0.26 (0.10–0.38)	0.16 (0.08–0.50)	0.532
IFN-γ (pg/mL)	0.39 (0.28–0.52)	0.47 (0.26–0.91)	0.411
VEGF-A (pg/mL)	194 (118–278)	173 (128–282)	0.933
IL-8 (pg/mL)	11.7 (9.7–15.0)	15.5 (10.1–28.3)	0.068
Neutrophils (N × 1000/µL)	5.15 (3.28–6.06)	NA	-
Lymphocytes (N × 1000/µL)	1.83 (1.52–2.32)	NA	-
Platelets (N × 1000/µL)	226 (190–266)	NA	-
NLR	2.44 (1.58–3.13)	NA	-
NPR	0.02 (0.01–0.03)	NA	-
Fibrinogen (mg/dL)	279 (245–350)	NA	-

IL: interleukin; ANG: angiopoietin; TNF: tumor necrosis factor; IFN: interferon; VEGF-A: vascular endothelial growth factor A; NLR: neutrophil-to-lymphocyte ratio; NPR: neutrophil-to-platelet ratio; NA: not available. Significant *p*-values are reported in bold.

**Table 4:** Spearman correlation analysis between serum lipids and cytokines levels in patients with schizophrenia (SCZ, n = 40).

		IL-13	TNF-α	IL-10	IL-8	IL-6	IL-5	IFN-γ	VEGF-A	ANG-2
**HDL cholesterol**	r_s_	0.082	0.089	−0.227	0.134	−0.180	0.155	0.065	−0.403	0.214
	*p* value	0.635	0.608	0.184	0.435	0.300	0.367	0.705	**0.015**	0.211
**Triglycerides**	r_s_	−0.043	0.153	0.257	−0.282	0.231	−0.216	0.110	0.240	−0.159
	*p* value	0.805	0.372	0.131	0.095	0.182	0.206	0.523	0.159	0.354
**Cholestanol**	r_s_	−0.144	0.010	−0.075	−0.273	−0.049	−0.144	0.093	−0.103	0.101
	*p* value	0.410	0.953	0.667	0.113	0.783	0.408	0.596	0.556	0.564
**Lathosterol**	r_s_	0.164	0.114	0.044	−0.292	−0.089	0.053	−0.031	0.169	−0.148
	*p* value	0.346	0.514	0.803	0.089	0.615	0.761	0.860	0.331	0.397
**Phytosterols**	r_s_	−0.216	−0.239	−0.067	−0.087	0.006	−0.169	0.108	−0.026	−0.219
	*p* value	0.214	0.166	0.702	0.619	0.973	0.331	0.538	0.880	0.206
**7-DHC**	r_s_	0.078	0.244	−0.232	0.189	0.128	0.092	−0.150	0.420	−0.217
	*p* value	0.657	0.157	0.179	0.278	0.470	0.601	0.391	**0.012**	0.211
**8-DHC**	r_s_	−0.038	0.054	−0.077	0.166	−0.057	0.084	−0.028	0.163	0.353
	*p* value	0.830	0.759	0.662	0.340	0.750	0.632	0.874	0.348	**0.037**

HDL: high-density lipoprotein; DHC: dehydrocholesterol; ANG: angiopoietin; IL: interleukin. Significant *p*-values are reported in bold.

**Table 5:** Linear regression analysis between antipsychotic doses (CPZeq) and discriminant variables in patients with schizophrenia (SCZ, n = 40).

Markers	Adjusted R^2^	F *	B	SE	beta	t	*p*-Value
HDL cholesterol	0.030	2.218	−0.013	0.009	−0.235	−1.489	0.145
Triglycerides	−0.008	0.690	−0.040	0.048	−0.134	−0.831	0.411
Lathosterol	−0.003	0.899	0.000	0.000	−0.154	−0.948	0.349
Phytosterols	−0.025	0.074	5 × 10^−5^	0.000	0.045	0.272	0.787
Cholestanol	−0.027	0.018	−4 × 10^−5^	0.000	−0.022	−0.136	0.893
7-DHC	0.052	3.083	0.000	0.000	0.277	1.756	0.087
8-DHC	0.076	4.125	0.000	0.000	0.317	2.031	**0.049**
ANG-2	−0.025	0.139	−0.203	0.546	−0.064	−0.373	0.712
IL-6	0.067	3.445	0.008	0.004	0.307	1.856	0.072
IL-10	0.165	7.904	0.006	0.002	0.434	2.811	**0.008**

* Regression degrees of freedom = 1, residual degrees of freedom = 38. SE: standard error; HDL: high-density lipoprotein; DHC: dehydrocholesterol; ANG: angiopoietin; IL: interleukin. Significant *p*-values are reported in bold.

## Data Availability

The raw data supporting the conclusions of this article will be made available by the authors on request, as the data are part of an ongoing study.

## Supplementary Materials

The following supporting information can be downloaded at https://www.mdpi.com/article/10.3390/ijms27167219/s1.

## Abbreviations

The following abbreviations are used in this manuscript:

SCZ	Schizophrenia
BBB	Blood–brain barrier
LDL	Low-density lipoprotein
HDL	High-density lipoprotein
CTRL	Healthy controls
DHC	Dehydrocholesterol
DHCR7	7-dehydrocholesterol reductase
IL	Interleukin
ANG	Angiopoietin
TNF	Tumor necrosis factor
IFN	Interferon
VEGF-A	Vascular endothelial growth factor A
TRS	Treatment-resistant schizophrenia

## References

[1] Charlson F.J., Ferrari A.J., Santomauro D.F., Diminic S., Stockings E., Scott J.G., McGrath J.J., A Whiteford H. (2018). Global epidemiology and burden of schizophrenia: Findings from the global burden of disease study 2016. Schizophr. Bull..

[2] Stahl S.M. (2018). Beyond the dopamine hypothesis of schizophrenia to three neural networks of psychosis: Dopamine, serotonin, and glutamate. CNS Spectr..

[3] Davison J., O’Gorman A., Brennan L., Cotter D.R. (2018). A systematic review of metabolite biomarkers of schizophrenia. Schizophr. Res..

[4] Zhang J., Liu Q. (2015). Cholesterol metabolism and homeostasis in the brain. Protein Cell.

[5] Gjerde P.B., Dieset I., Simonsen C., Hoseth E.Z., Iversen T., Lagerberg T.V., Lyngstad S.H., Mørch R.H., Skrede S., Andreassen O.A. (2018). Increase in serum HDL level is associated with less negative symptoms after one year of antipsychotic treatment in first-episode psychosis. Schizophr. Res..

[6] Wójciak P., Domowicz K., Rybakowski J.K. (2021). Metabolic indices in schizophrenia: Association of negative symptoms with higher HDL cholesterol in female patients. World J. Biol. Psychiatry.

[7] Chen L., Zheng C., Luan H., Chen X. (2024). Clinical and diagnostic value of high-density lipoprotein-based inflammatory indices and lipid ratios in young adults with schizophrenia. J. Inflamm. Res..

[8] Radford-Smith D.E., Yates A.G., Rizvi L., Anthony D.C., Probert F. (2023). HDL and LDL have distinct, opposing effects on LPS-induced brain inflammation. Lipids Health Dis..

[9] Giannattasio A., Castaldo A., Grieco M., Gelzo M., Cernera G., Castaldo G., Tipo V. (2025). Serum HDL and their subfractions are impaired in multisystem inflammatory syndrome in children (MIS-C). J. Transl. Med..

[10] Pereira S., Au E., Agarwal S.M., Wright D.C., Hahn M.K. (2023). Antipsychotic-induced alterations in lipid turnover. Endocrinology.

[11] Rogdaki M., McCutcheon R.A., D’Ambrosio E., Mancini V., Watson C.J., Fanshawe J.B., Carr R., Telesia L., Martini M.G., Philip A. (2024). Comparative physiological effects of antipsychotic drugs in children and young people: A network meta-analysis. Lancet Child Adolesc. Health.

[12] Korade Ž., Liu W., Warren E.B., Armstrong K., Porter N.A., Konradi C. (2017). Effect of psychotropic drug treatment on sterol metabolism. Schizophr. Res..

[13] Korade Ž., Anderson A., Balog M., Tallman K.A., Porter N.A., Mirnics K. (2023). Chronic aripiprazole and trazodone polypharmacy effects on systemic and brain cholesterol biosynthesis. Biomolecules.

[14] Gelzo M., Iacotucci P., Sica C., Liguori R., Comegna M., Carnovale V., Russo A.D., Corso G., Castaldo G. (2020). Influence of pancreatic status on circulating plasma sterols in patients with cystic fibrosis. Clin. Chem. Lab. Med..

[15] Corso G., Gelzo M., Barone R., Clericuzio S., Pianese P., Nappi A., Russo A.D. (2011). Sterol profiles in plasma and erythrocyte membranes in patients with Smith-Lemli-Opitz syndrome: A six-year experience. Clin. Chem. Lab. Med..

[16] Asztalos B.F., Tani M., Schaefer E.J. (2011). Metabolic and functional relevance of HDL subspecies. Curr. Opin. Lipidol..

[17] Ballout R.A., Kong H., Sampson M., Otvos J.D., Cox A.L., Agbor-Enoh S., Remaley A.T. (2021). The NIH Lipo-COVID Study: A Pilot NMR Investigation of Lipoprotein Subfractions and Other Metabolites in Patients with Severe COVID-19. Biomedicines.

[18] Korade Z., Xu L., Shelton R., Porter N.A. (2010). Biological activities of 7-dehydrocholesterol-derived oxysterols: Implications for Smith-Lemli-Opitz syndrome. J. Lipid Res..

[19] Solberg D.K., Bentsen H., Refsum H., Andreassen O.A. (2015). Association between serum lipids and membrane fatty acids and clinical characteristics in patients with schizophrenia. Acta Psychiatr. Scand..

[20] Mhalla A., Salah W.B.H., Mensi R., Amamou B., Messaoud A., Gassab L., Douki W., Najjar M.F., Gaha L. (2018). Lipid profile in schizophrenia: Case control study. Tunis. Med..

[21] Colvin P.L., Parks J.S. (1999). Metabolism of high density lipoprotein subfractions. Curr. Opin. Lipidol..

[22] Williams J.A., Burgess S., Suckling J., Lalousis P.A., Batool F., Griffiths S.L., Palmer E., Karwath A., Barsky A., Gkoutos G.V. (2022). Inflammation and brain structure in schizophrenia and other neuropsychiatric disorders: A mendelian randomization study. JAMA Psychiatry.

[23] Labonté C., Zhand N., Park A., Harvey P.D. (2022). Complete blood count inflammatory markers in treatment-resistant schizophrenia: Evidence of association between treatment responsiveness and levels of inflammation. Psychiatry Res..

[24] Gelzo M., Granato G., Albano F., Arcucci A., Dello Russo A., De Vendittis E., Ruocco M.R., Corso G. (2014). Evaluation of cytotoxic effects of 7-dehydrocholesterol on melanoma cells. Free Radic. Biol. Med..

[25] Liu W., Xu L., Lamberson C.R., Merkens L.S., Steiner R.D., Elias E.R., Haas D., Porter N.A. (2013). Assays of plasma dehydrocholesteryl esters and oxysterols from Smith-Lemli-Opitz syndrome patients. J. Lipid Res..

[26] Björkhem I. (2006). Crossing the barrier: Oxysterols as cholesterol transporters and metabolic modulators in the brain. J. Intern. Med..

[27] Scholz A., Plate K.H., Reiss Y. (2015). Angiopoietin-2: A multifaceted cytokine that functions in both angiogenesis and inflammation. Ann. N. Y. Acad. Sci..

[28] Schwarz E., van Beveren N.J., Ramsey J., Leweke F.M., Rothermundt M., Bogerts B., Steiner J., Guest P.C., Bahn S. (2014). Identification of subgroups of schizophrenia patients with changes in either immune or growth factor and hormonal pathways. Schizophr. Bull..

[29] Thurston G., Rudge J.S., Ioffe E., Zhou H., Ross L., Croll S.D., Glazer N., Holash J., McDonald D.M., Yancopoulos G.D. (2000). Angiopoietin-1 protects the adult vasculature against plasma leakage. Nat. Med..

[30] Yancopoulos G.D., Davis S., Gale N.W., Rudge J.S., Wiegand S.J., Holash J. (2000). Vascular-specific growth factors and blood vessel formation. Nature.

[31] American Psychiatric Association, DSM-5 Task Force (2013). Diagnostic and Statistical Manual of Mental Disorders: DSM-5™.

[32] Kay S.R., Fiszbein A., Opler L.A. (1987). The positive and negative syndrome scale (PANSS) for schizophrenia. Schizophr. Bull..

[33] Howes O.D., McCutcheon R., Agid O., de Bartolomeis A., van Beveren N.J., Birnbaum M.L., Bloomfield M.A., Bressan R.A., Buchanan R.W., Carpenter W.T. (2017). Treatment-Resistant Schizophrenia: Treatment Response and Resistance in Psychosis (TRRIP) Working Group Consensus Guidelines on Diagnosis and Terminology. Am. J. Psychiatry.

[34] Gelzo M., Clericuzio S., Barone R., D’Apolito O., Dello Russo A., Corso G. (2012). A routine method for cholesterol and 7-dehydrocholesterol analysis in dried blood spot by GC-FID to diagnose the Smith-Lemli-Opitz syndrome. J. Chromatogr. B.

[35] Gelzo M., Caputo M., Comegna M., Cernera G., Di Minno A., Scialo F., Castaldo G., Corso G. (2021). Exploiting sterol profile analysis: Over ten years of experience. A narrative review. Acta Med. Mediterr..

